# The Effects of Remifentanil and Fentanyl on Emergence Agitation in Pediatric Strabismus Surgery

**DOI:** 10.3390/children9050606

**Published:** 2022-04-24

**Authors:** Jongyoon Baek, Sang Jin Park, Jun Oh Kim, Minhyun Kim, Do Young Kim, Eun Kyung Choi

**Affiliations:** Department of Anesthesiology and Pain Medicine, Yeungnam University College of Medicine, 170, Hyeonchung-ro, Nam-gu, Daegu 42415, Korea; jongyoonbaek@gmail.com (J.B.); apsj0718@naver.com (S.J.P.); mongsika@naver.com (J.O.K.); minhyun103@gmail.com (M.K.); maiden_blush@daum.net (D.Y.K.)

**Keywords:** emergence agitation, sevoflurane, remifentanil, fentanyl, anesthesia

## Abstract

Emergence agitation (EA) is one of the main concerns in the field of pediatric anesthesia using sevoflurane. We investigated the effects of remifentanil and fentanyl on the incidence of EA in pediatric patients undergoing strabismus surgery. Ninety children were randomly allocated into two groups and received either remifentanil (group R: intraoperatively remifentanil 0.2 μg/kg/min) or fentanyl (group F: fentanyl 2 μg/kg at anesthetic induction) intraoperatively. After surgery, EA incidence was assessed using a four-point agitation scale and Pediatric Anesthesia Emergence Delirium (PAED) scale in the post-anesthesia care unit. Face, leg, activity, cry, and consolability (FLACC) scores for postoperative pain were also assessed. The incidence of EA using the four-point agitation scale (scores ≥ 3) was similar in both groups (remifentanil group, 28.89% vs. fentanyl group, 24.44%). Similar results were obtained using the PAED scale (scores > 12), with an incidence of 33.33% in the remifentanil group and 26.67% in the fentanyl group. Differences in FLACC scores were not found to be statistically significant. A single bolus administration of fentanyl during anesthetic induction and continuous infusion of remifentanil during surgery had similar effects on the EA incidence in these pediatric patients.

## 1. Introduction

Emergence agitation (EA) is one of the main concerns in the field of pediatric anesthesia using short-acting volatile anesthetics, such as sevoflurane [1]. This postanesthetic behavioral disturbance is frequently developed in ophthalmological surgery [2], and EA may cause harm to the child and distress to caregivers, leading to dissatisfaction with the anesthetic. Among the various pharmacotherapies and optimal anesthesia techniques used to reduce EA, opioids are popular and useful agents for the prevention and/or treatment of EA.

Fentanyl and remifentanil are short-acting opioids that are widely used in pediatric patients. Both drugs have been used as sedatives and analgesics, which can affect the hemodynamic response of blunt surgical stimulation as well as tracheal intubation. Recently, the clinical usefulness of these drugs has been suggested to reduce EA occurring post sevoflurane anesthesia [3,4]. However, opioids still have some adverse effects on respiratory depression, postoperative vomiting, and opioid-induced hyperalgesia during the postoperative period.

In this regard, we studied the effects of single bolus administration of fentanyl during anesthetic induction and continuous remifentanil infusion during surgery on the incidence of EA in pediatric patients undergoing strabismus surgery. Additionally, we compared the recovery characteristics, including respiratory depression, postoperative vomiting, and postoperative pain.

## 2. Materials and Methods

### 2.1. Study Design and Protocol

This study was approved by the Institutional Review Board of our institution (YUMC2017-04-071) and was registered on ClinicalTrials.gov (NCT03807011). Written informed consent was obtained from all the children’s parents. Children with the American Society of Anesthesiologists physical status classification I or II and aged 2–8 years were scheduled for strabismus surgery. Children with a history of airway problems, recent respiratory tract infection, cognitive impairment, or psychological disease were excluded from this study.

Using computer-generated randomization and an opaque sealed envelope, children were randomly allocated to one of the two following groups: remifentanil group (group R; intraoperatively remifentanil 0.2 μg/kg/min) and fentanyl group (group F; fentanyl 2 μg/kg at anesthetic induction). The patients received ketamine 1 mg/kg intravenously in the waiting room. After arriving at the operating room, we monitored electrocardiography, pulse oximetry, and blood pressure. Anesthesia was induced using sevoflurane (3–3.5 vol%) and rocuronium (0.8 mg/kg). During induction, remifentanil was continuously infused at a rate of 0.2 μg/kg/min until the end of surgery (group R), or a bolus dose of fentanyl (2 μg/kg) was administered intravenously (group F). Anesthesia was maintained using sevoflurane 2–3 vol% and oxygen with 50% air, and proper hemodynamic stability was maintained within 20% of pre-induction blood pressure and heart rate, and a bispectral value of 40–60. Vital signs were monitored periodically during the surgery, the incidence of oculocardiac reflex (OCR; 20% decline in heart rate after traction of extraocular muscle) was observed, and then the surgeon stopped the traction. If the heart rate was less than 60, or OCR persists, 0.01 mg/kg atropine was administered. To consider the side effect of opioids, intraoperative bradycardia (more than 20% decline compared to preinduction value) was recorded separately from the OCR. At the end of the surgery, sevoflurane was discontinued and antagonism of muscle relaxation was performed using pyridostigmine and glycopyrrolate. All patients received ketorolac 0.5 mg/kg for postoperative pain control. Tracheal extubation was done when the patient had a spontaneous breath and purposeful movement, and extubation time (from the cessation of sevoflurane to the removal of the endotracheal tube) was recorded.

In the post-anesthetic care unit (PACU), the parents were allowed to be present besides their children. EA was assessed using the four-point agitation scale [5] and the Pediatric Anesthesia Emergence Delirium (PAED) scale [6] at 5-, 10-, 15-, and 30-min. Children were considered agitated with a four-point agitation scale score ≥3 [7] or a PAED scale score >12 [8]. If the PAED score was >15 (severe agitation) [9], intravenous fentanyl 1 μg/kg was administered. Face, legs, activity, cry, and consolability (FLACC) scores for postoperative pain assessment [10] and sedation scores using the University of Michigan Sedation Scale (0–4) [11] were also assessed at the same time points. The incidence of nausea and vomiting was recorded, and rescue antiemetics (ramosetron 6 mcg/kg) was administered when the patient experienced vomiting. Additionally, respiratory depression (SpO2 < 95%) was also evaluated. All values were assessed by an anesthesiologist who was blinded to the study protocol.

### 2.2. Statistical Analysis

In a preliminary study, the incidence of EA after strabismus surgery was 60%. A sample size using a power analysis (α = 0.05, β = 0.2) was calculated; 42 patients per group were required to detect a 50% reduction in the incidence of EA (from 60% to 30%). Assuming a dropout rate of < 10%, 94 patients were recruited. All statistical analyses were performed using SPSS version 23.0 software (IBM Corporation, Armonk, NY, USA) and results were expressed as mean ± standard deviation, number (%), median (interquartile range), and 95% confidence intervals. Student’s *t*-test, Chi-square test, Fisher’s exact test, or Mann-Whitney U test were used as appropriate. Statistical significance was set at *p* < 0.05.

## 3. Results

Of the 94 children who underwent the screening test, three were excluded due to withdrawal of consent and one owing to upper respiratory tract infection. The data on age, sex, weight, operation time, anesthesia time, and extubation time were similar between the two groups (Table 1). There was no significant difference in the four-point agitation scale and PAED scores in either group at any time point (5, 10, 15, and 30 min after arrival in the PACU; Table 2). The incidence of EA using the four-point agitation scale (scores ≥ 3) was similar in both groups (group R, 28.89% vs. group F, 24.44%). The incidence of EA using the PAED scale (scores > 12) was also similar in both the groups (group R, 33.33% vs. group F, 26.67%) (Table 3). The FLACC scores between the groups were not statistically significant (Table 4). No difference was observed in the sedation scores (Table 5) or incidence of perioperative adverse outcomes in either group (Table 6).

## 4. Discussion

A single bolus administration of fentanyl during anesthetic induction and continuous infusion of remifentanil during surgery had similar effects on the EA incidence in children undergoing strabismus surgery. Additionally, postoperative sedation and postoperative pain scores were similar between the two opioids.

EA is commonly seen in children during the early post-anesthesia period following general anesthesia. Several factors can affect the incidence of EA, including age, type of anesthetics, type of surgery, preoperative anxiety, and postoperative pain. Among these factors, EA is more common at a young age [12], and when sevoflurane is used during general anesthesia [13]. In particular, strabismus surgery under sevoflurane has reported increased rates of EA [2], with up to 80% in children [14]. Children in this study had several risk factors for EA: younger preschool age, ophthalmic surgery, and use of sevoflurane. However, the incidence of EA showed a lower tendency compared to the results of previous studies. Besides the positive effects of both experimental drugs on EA, this might be because of parental presence during the PACU stay, which might be allowed to attenuate the provocation of EA due to an unfamiliar environment and disturbed vision when awakening from anesthesia. In children, patient anxiety and recovery in a strange situation can cause EA. Therefore, parental presence during anesthetic induction and/or emergence is expected to help reduce EA [15,16]. Additionally, we administered low-dose ketamine before entering the operating room considering the difficulty of being separated from their parents. Many studies have shown its preventative effect on reducing EA [17,18], however, they were controversial [19,20]. Its beneficial effects on EA have been shown mostly when it was administered before the end of surgery rather than prior to anesthetic induction [17,18]. However, it is unclear ketamine’s effect on EA in our study, although administration of ketamine might not affect the differences inf inter-group incidence of EA. Postoperative pain is another risk factor for EA [12]. Therefore, adequate postoperative analgesia (ketorolac administration before the end of surgery) might affect the overall incidence of EA.

Fentanyl is a short-acting opioid that possesses synthetic μ receptor–stimulating properties and is widely used in children as an intraoperative sedative and analgesic. Fentanyl has a short peak time and action duration after intravenous administration [21]; however, like other opioids, it has potential negative effects through central nervous system actions [21]. Concerning the prevention of EA, a meta-analysis showed that fentanyl (1 μg/kg) around the end of surgery can decrease the EA incidence after general anesthesia [22]; however, this protocol revealed an increased incidence of postoperative nausea and prolonged PACU stay. Therefore, we selected earlier administration of fentanyl (during intubation) considering hemodynamic stability during tracheal intubation, intraoperative analgesia, and postoperative complications. In several previous studies, fentanyl during anesthetic induction (2–2.5 μg/kg) reduced the EA incidence after sevoflurane anesthesia without significant adverse events [23,24]. In this study, EA occurrence was 24.44% on the four-point agitation scale and 26.67% on the PAED scale when fentanyl 2 μg/kg was used, which was compatible with reduced EA in children.

Similar to fentanyl, remifentanil has been suggested to decrease the EA incidence followed by sevoflurane anesthesia [4,25]. Unlike conventional opioids, remifentanil has an ester linkage, and these unique pharmacokinetics allow for more rapid onset and offset [26]. In addition, a short context-sensitive half-life of remifentanil allowed for intraoperative continuous infusion [27]. However, postoperative pain might develop with the use of remifentanil owing to its faster elimination properties. A recent meta-analysis regarding remifentanil showed faster recovery, less respiratory depression, and higher analgesic requirements during the immediate postoperative period [26]. In this study, intraoperative continuous remifentanil infusion (0.2 μg/kg/min) was associated with a decrease in the EA incidence. A report showed that intraoperative remifentanil (0.1 μg/kg/min) reduced the EA incidence in children undergoing adenotonsillectomy with sevoflurane anesthesia [4]. In a study by Choi et al., remifentanil at the rate of 0.1 μg/kg/min reduced EA in children aged 3–9 years undergoing strabismus surgery under sevoflurane general anesthesia [25]. The mechanism of fentanyl and remifentanil reducing EA is unclear, one possible explanation is the sevoflurane sparing effect. Remifentanil reduces the inhaled concentration of sevoflurane dose-dependently in children [28]. Another possible mechanism is the increased analgesic properties of both drugs in reducing EA, although it has a short half-life.

To date, however, there have been no studies comparing the efficacy of fentanyl and remifentanil on EA from sevoflurane. In this study, the incidence of EA was similarly low in both groups in children who underwent sevoflurane anesthesia without differences in complications (fentanyl 24.44% vs. remifentanil 28.89% on the four-point agitation scale, fentanyl 26.67% vs. remifentanil 33.33% on the PAED scale). From the above similar results of EA for both fentanyl and remifentanil, it might be interpreted that both regimens (single bolus fentanyl at anesthetic induction and continuous infusion of remifentanil during surgery) were effective in high-risk patients with EA.

As mentioned above, postoperative pain can be a contributing factor to EA [12]. It is difficult to completely eliminate the pain effect on the diagnosis of EA as the distinction of behaviors related to either EA or postoperative pain may be obscure in younger patients. Therefore, we administered ketorolac 0.5 mg/kg to all children with an expected attenuation of at least the contribution of surgical pain. In addition to EA scores, FLACC scores in the PACU were similar in both groups; however, it is conceivable that the effects of postoperative pain might be of little impact on the difference in the occurrence of EA. Moreover, preventative ketorolac administration might offset the pain difference, if it should be used, in both groups; this can be explained as a minor stimulating procedure of ophthalmic surgery that causes less pain. Additionally, a common concern regarding the use of remifentanil is remifentanil-induced hyperalgesia [27]. In many studies, patients receiving remifentanil, compared to other opioids, had a postoperative high pain score and high analgesic requirements [29,30]. A practicable explanation is based on remifentanil-induced hyperalgesia, which could be associated with high doses and long-term remifentanil anesthesia. In this study, we administered 0.2 μg/kg/min of remifentanil; we could not find higher tolerance and/or higher hyperalgesia compared to fentanyl anesthesia.

A few limitations of this study were as follows. First, although we selected the optimal dose and administration time of both the drugs based on previous studies demonstrating the efficacy of EA, regimens of fentanyl and remifentanil might not be equipotent. Second, is that although we used a validated method of EA measurement by a single investigator, the assessment of EA could still be subjective. Therefore, more reliable assessment scales may be required in future studies. Finally, we administered the low dose ketamine preoperatively to reduce separation anxiety, however, it might be a bias on the incidence of EA in both groups, rather than inter-group differences. Further study would be required to rule out the potential role of ketamine on EA.

In conclusion, 0.2 μg/kg/min remifentanil during surgery and fentanyl 2 μg/kg of fentanyl administration at anesthetic induction during ophthalmic surgery had similar rates of EA, postoperative pain scores, and other emergence characteristics (respiratory depression, nausea, and vomiting).

## Figures and Tables

**Table 1:** Demographics and anesthetic data.

Variable	Group R (*n* = 45)	Group F (*n* = 45)	*p*-Value
Age (years)	6.24 ± 1.09	5.73 ± 1.37	0.058
Sex (male/female)	25 (55.6)/20 (44.4)	25 (55.6)/20 (44.4)	1.000
Weight (kg)	22.82 ± 3.46	21.91 ± 4.36	0.278
Operation time (minutes)	41.67 ± 10.06	44.56 ± 12.61	0.288
Anesthesia time (minutes)	64.11 ± 11.55	67.22 ± 12.68	0.259
Extubation time (minutes)	10.76 ± 2.41	9.84 ± 2.96	0.058

Values were presented as mean ± SD (standard deviation)/number (percent). Group R, continuous infusion of remifentanil 0.2 μg/kg/min during surgery; Group F, bolus injection of fentanyl 2 μg/kg at anesthetic induction.

**Table 2:** Scores of emergence agitation at four time points in the post-anesthetic care unit.

EA Scale	Group R (*n* = 45)	Group F (*n* = 45)	Median Difference	95% CI	*p*-Value
FPAS score					
at 5 min in PACU	2(2–3)	2 (2–2)	0	(−0.001, 0.001)	0.810
at 10 min in PACU	2 (2–2)	2 (2–2)	0	(−0.001, 0.001)	0.537
at 15 min in PACU	2 (2–2)	2 (2–2)	0	(−0.001, 0.001)	0.647
at 30 min in PACU	2 (1–2)	2 (1–2)	0	(−0.001, 0.001)	0.319
PAED score					
at 5 min in PACU	12 (11–12)	11 (11–12)	0	(−0.001, 0.999)	0.611
at 10 min in PACU	11 (10–13)	11 (10–12)	0	(−0.001, 0.999)	0.416
at 15 min in PACU	10 (9–10)	10 (9–10)	0	(−0.001, 0.001)	0.949
at 30 min in PACU	8 (8–9)	8 (7–9)	0	(−0.001, 0.999)	0.072

Values were presented as median (IQR, interquartile range). Group R, continuous infusion of remifentanil 0.2 μg/kg/min during surgery; Group F, bolus injection of fentanyl 2 μg/kg at anesthetic induction; PACU, post anesthetic care unit; CI, confidence interval; FPAS, four-point agitation scale; PAED, Pediatric Anesthesia Emergence Delirium.

**Table 3:** The incidence of emergence agitation.

EA Scale	Group R (*n* = 45)	Group F (*n* = 45)	OR	95% CI for OR	*p*-Value
FPAS score ≥ 3	13 (28.89)	11 (24.44)	0.796	(0.312, 2.032)	0.634
PAED score > 12	15 (33.33)	12 (26.67)	0.727	(0.294, 1.799)	0.490
PAED score > 15 (severer EA)	0 (0)	0 (0)	-	-	-

Values were presented as median (IQR, interquartile range). Group R, continuous infusion of remifentanil 0.2 μg/kg/min during surgery; Group F, bolus injection of fentanyl 2 μg/kg at anesthetic induction; PACU, post anesthetic care unit; CI, confidence interval; FPAS, four-point agitation scale; PAED, Pediatric Anesthesia Emergence Delirium.

**Table 4:** Face, legs, activity, cry, and consolability (FLACC) scores.

FLACC Score	Group R (*n* = 45)	Group F (*n* = 45)	Median Difference	95% CI	*p*-Value
at 5 min in PACU	6 (6–6)	6 (5–6)	0	(−0.001, 0.999)	0.061
at 10 min in PACU	6 (6–6)	6 (5–6)	0	(−0.001, 0.999)	0.116
at 15 min in PACU	5 (5–6)	5 (5–6)	0	(−0.001, 0.001)	0.655
at 30 min in PACU	5 (5–6)	6 (5–6)	0	(−0.001, 0.001)	0.145

Values were presented as median (IQR, interquartile range). Group R, continuous infusion of remifentanil 0.2 μg/kg/min during surgery; Group F, bolus injection of fentanyl 2 μg/kg at anesthetic induction; PACU, post anesthetic care unit; CI, confidence interval.

**Table 5:** University of Michigan Sedation Scale scores.

Sedation Score	Group R (*n* = 45)	Group F (*n* = 45)	Median Difference	95% CI	*p*-Value
at 5 min in PACU	0 (0–0)	0 (0–1)	0	−0.001, 0.001	0.215
at 10 min in PACU	0 (0–0)	0 (0–0)	0	-	1.000
at 15 min in PACU	0 (0–0)	0 (0–0)	0	-	1.000
at 30 min in PACU	0 (0–0)	0 (0–0)	0	-	1.000

Values were presented as median (IQR, interquartile range). Group R, continuous infusion of remifentanil 0.2 μg/kg/min during surgery; Group F, bolus injection of fentanyl 2 μg/kg at anesthetic induction; PACU, post anesthetic care unit; CI, confidence interval.

**Table 6:** Perioperative adverse outcomes.

Variable	Group R (*n* = 45)	Group F (*n* = 45)	OR	95% CI for OR	*p*-Value
Intraoperative bradycardia	0 (0)	0 (0)	-	-	-
Oculocardiac reflex	3 (6.67)	5 (11.11)	1.750	(0.392, 7.807)	0.459
Use of atropine	1 (2.22)	1 (2.22)	1.000	(0.061, 16.496)	1.000
Desaturation	0 (0)	0 (0)	-	-	-
Nausea/Vomiting	1 (2.22)	0 (0)	-	-	-

Group R, continuous infusion of remifentanil 0.2 μg/kg/min during surgery; Group F, bolus injection of fentanyl 2 μg/kg at anesthetic induction; OR, odds ratio; CI, confidence interval.

## Data Availability

The data presented in this study are available on request from the corresponding author.
